# Consensus Modeling for Prediction of Estrogenic Activity of Ingredients Commonly Used in Sunscreen Products

**DOI:** 10.3390/ijerph13100958

**Published:** 2016-09-29

**Authors:** Huixiao Hong, Diego Rua, Sugunadevi Sakkiah, Chandrabose Selvaraj, Weigong Ge, Weida Tong

**Affiliations:** 1 Division of Bioinformatics and Biostatistics, National Center for Toxicological Research, U.S. Food and Drug Administration, 3900 NCTR Road, Jefferson, AR 72079, USA; Suguna.Sakkiah@fda.hhs.gov (S.S.); Selvaraj.Chandrabose@fda.hhs.gov (C.S.); Weigong.Ge@fda.hhs.gov (W.G.); Weida.Tong@fda.hhs.gov (W.T.); 2 Division of Nonprescription Drug Products, Center for Drug Evaluation and Research, U.S. Food and Drug Administration, 10903 New Hampshire Avenue, Silver Spring, MD 20993, USA

**Keywords:** sunscreen, ingredient, estrogenic activity, prediction, model

## Abstract

Sunscreen products are predominantly regulated as over-the-counter (OTC) drugs by the US FDA. The “active” ingredients function as ultraviolet filters. Once a sunscreen product is generally recognized as safe and effective (GRASE) via an OTC drug review process, new formulations using these ingredients do not require FDA review and approval, however, the majority of ingredients have never been tested to uncover any potential endocrine activity and their ability to interact with the estrogen receptor (ER) is unknown, despite the fact that this is a very extensively studied target related to endocrine activity. Consequently, we have developed an in silico model to prioritize single ingredient estrogen receptor activity for use when actual animal data are inadequate, equivocal, or absent. It relies on consensus modeling to qualitatively and quantitatively predict ER binding activity. As proof of concept, the model was applied to ingredients commonly used in sunscreen products worldwide and a few reference chemicals. Of the 32 chemicals with unknown ER binding activity that were evaluated, seven were predicted to be active estrogenic compounds. Five of the seven were confirmed by the published data. Further experimental data is needed to confirm the other two predictions.

## 1. Introduction 

Endocrine active chemicals arise from many different sources, including pesticides, industrial chemicals, pharmaceuticals, and consumer products. Exposure to any of these chemicals may systemically mimic the biological activities of hormones. Since the mid-1990s, there has been public concern about endocrine disrupting chemicals and this concern is made more burdensome by continuous ingredient innovations. Catching up via chemical hazard screening approaches has been actively pursued, but focused on industrial, pharmaceutical and agricultural chemicals due to their potential acute toxicity as well as greater exposure in humans. Relatively less attention has been paid to cosmetic and personal care products, despite the significant innovations witnessed in this industry. 

Topically-applied products intended to help prevent sunburn, decrease the risk of skin cancer, or decrease the effects of skin aging caused by the sun are collectively considered to be “sunscreen products”. In the United States (US), these products are regulated by the Food & Drug Administration (FDA) as drugs. Sunscreen products currently available in the US are predominantly regulated as over-the-counter (OTC) drugs. The ingredient workhorses in sunscreen products are the “active” ingredients which function as ultraviolet (UV) filters of either UVA and/or UVB rays. The OTC drug review involves a determination by FDA that a sunscreen UV filter is generally recognized as safe and effective (GRASE) based on the available scientific evidence. Once an ingredient UV filter receives GRASE status via an OTC drug review process, new formulations using these ingredients do not require FDA premarket review and approval. The latest OTC monograph setting FDA’s rules for sunscreen products was published in the Federal Register on 17 June 2011 [1] and instituted fully in December of 2013.

There are risks and benefits associated with sunscreen use. The benefit is to lessen the chances of developing skin cancer when used as directed with other sun protection measures. FDA and other public health agencies continue to urge consumers to take sun protection measures, of which regular use of broad spectrum sunscreen products with a minimum SPF value of 15 is one element [2]. The risk is with continuous innovation in sunscreen formulation technology and its influence on the market use of sunscreen UV filters as well as excipients, which are typically not rigorously tested. Such is the case when assessing the risk of potential adverse effects deriving from ingredient endocrine activity. Even the ability of an ingredient to interact with the estrogen receptor (ER), which is a very extensively studied target related to endocrine activity, may be unknown. Therefore, a rapid way to categorize ingredients with unknown estrogenic activity into active and inactive ones is deemed helpful to provide a basis for prioritizing chemicals for more definitive but expensive testing. 

To evaluate the estrogenic activity of UV filters used in sunscreen products worldwide, we searched against in the publically available database referred to as the endocrine disruptor knowledge base (EDKB). EDKB actually contains in vitro and in vivo experimental estrogenic data for more than 3000 chemicals from multiple assays and its structures [3]. Since most of UV filters ingredients were found to have no such experimental data, we then used a consensus modeling method to predict their qualitatively and quantitatively binding activity towards the estrogen receptor (ER). The consensus modeling comprised two Decision Forest (DF) models that were built using two different training data sets. The two DF models were validated using five-fold cross validations and external chemicals. In addition to utilizing this consensus modeling to predict ER binding activity of UV filters, similar predictions were done on unrelated compounds to make reference comparisons as well to a few excipient ingredients frequently added to sunscreen formulations. 

## 2. Materials and Methods 

### 2.1. Study Design

**Figure 1:**
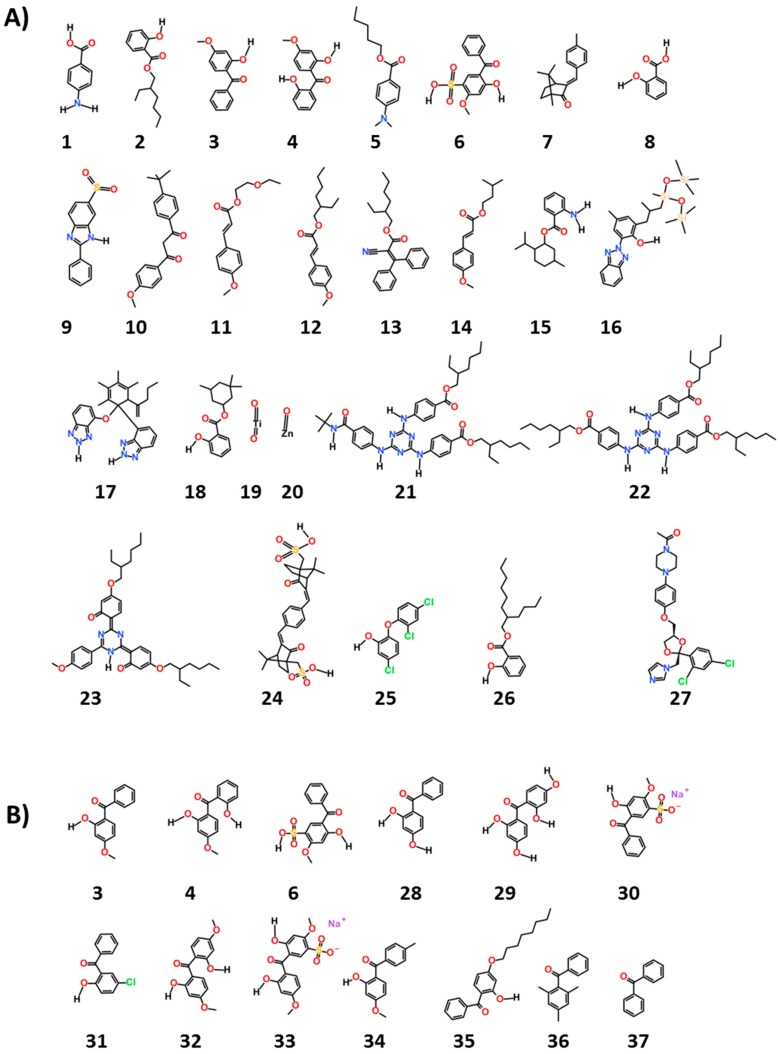
Structures of the 38 ingredients selected for this work. The numbers under structures were used in the text and Tables. The compounds shown in panel (**A**) were used in UV and non-UV filers; The compounds given in panel (**B**) are the benzophenone derivatives (potential excipients in sunscreen products and cosmetics).

We chose 38 ingredients of interest and their structures are given in Figure 1. We searched experimental estrogenic activity data for these ingredients on the estrogenic activity database (EADB) [4], a recently updated database in the endocrine disruptor knowledge base (EDKB) [3]. 

**Figure 2:**
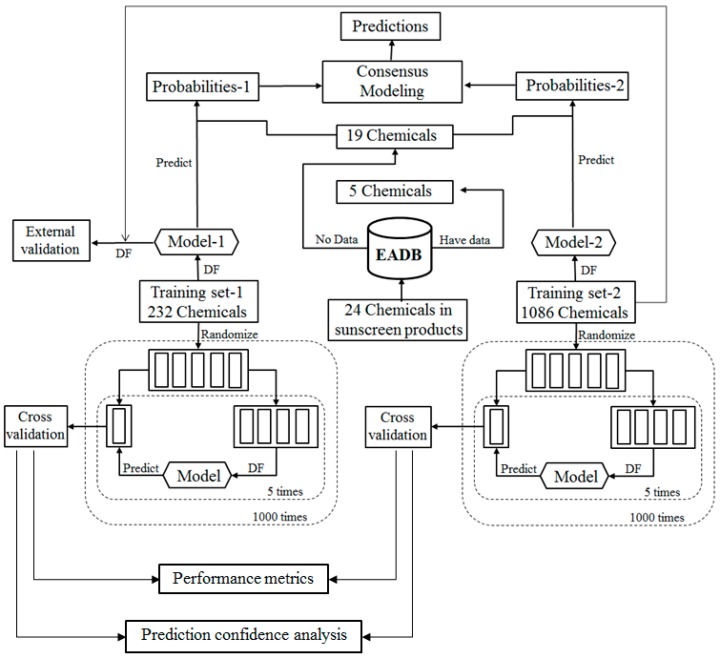
Workflow of the consensus classification modeling for classifying sunscreen ingredients as ER binders and non-binders.

**Figure 3:**
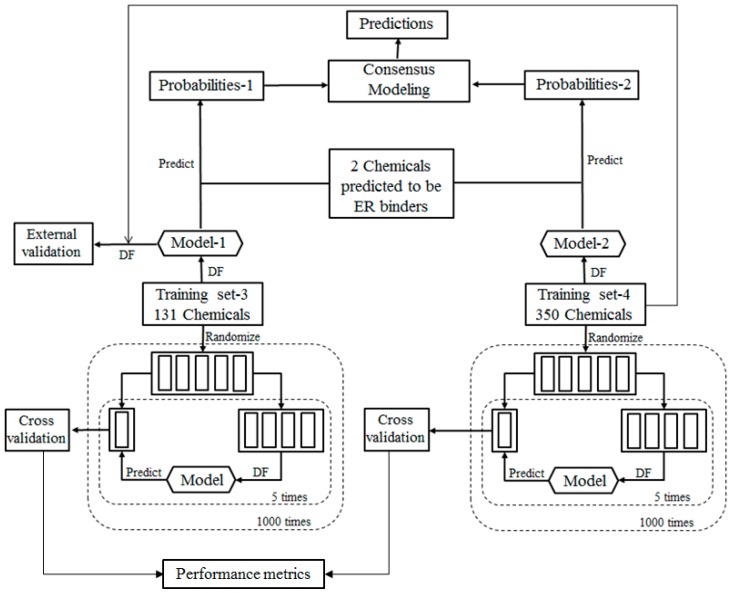
Workflow of the consensus regression modeling for estimating ER binding affinity of sunscreen ingredients.

For the ingredients that were not contained in EABD, their estrogenic activities were predicted using consensus DF models in two tiers. In the first tier, the ingredients were qualitatively classified as ER binders and non-binders as illustrated by the workflow shown in Figure 2. In the second tier, ER binding affinities of the sunscreen ingredients that were classified as ER binders in the first tier prediction were estimated using a quantitative consensus regression model as depicted by Figure 3.

The qualitative consensus classification model development is illustrated by the workflow shown in Figure 2. The 232 chemicals with ER binding activity determined in our laboratory [5] were used as a training data set (TS-1) to build a DF classification model (M-1). The 1086 chemicals with estrogenic activity data curated in our previous study [6] were used as another training data set (TS-2) to construct another DF classification model (M-2). To demonstrate, reliable individual DF classification models were developed using TS-1 and TS-2, 1000 iterations of 5-fold cross validations were carried out using TS-1 and TS-2. Most chemicals in TS-2 were not included in TS-1 and thus were used as an external validation set to estimate performance of the classification model M-1 that was trained using TS-1. The individual DF classification models (M-1 and M-2) were then used for classification of the ingredients lacking experimental data into ER binder or non-binder. The classification models output probabilities represent the likelihood of the ingredients to be classified as ER binders. Two probabilities of a compound were then averaged as a consensus classification of the ingredient as an ER binder or non-binder.

For the ingredients that were classified as potential ER binders, their binding affinities to ER were then qualitatively estimated using a consensus DF regression model. The quantitative consensus DF regression model development is illustrated by the workflow shown in Figure 3. The logarithmic relative binding affinity (logRBA) values of 131 ER binders from TS-1 were used as a training data set (TS-3) to build a DF regression model (M-3). The 350 chemicals in TS-2 are ER binders with logRBA values experimentally determined and were used as another training data set (TS-4) to construct another DF regression model (M-4). To demonstrate reliable individual DF regression models can be developed using TS-3 and TS-4, 1000 iterations of 5-fold cross validations were carried out using TS-3 and TS-4. Most chemicals in TS-4 were not included in TS-3 and thus were used as an external validation set to estimate the performance of DF regression model M-3 that was trained using TS-3. The individual DF regression models (M-3 and M-4) were then used for estimating logRBA values for the sunscreen ingredients that were classified as ER binders. For each ingredient, the two logRBA values output from M-3 and M-4 were then averaged for a consensus prediction of estrogenic activity for the ingredient.

### 2.2. Training Data Sets

Two data sets (TS-1 and TS-2) were used for training qualitative DF classification models and two data sets (TS-3 and TS-4) were used for training quantitative DF regression models. The data sets contain the ER binding activity measured using the competitive rat ER binding assay in our previous studies [6] and can be obtained from our databases EADB and EDKB [3,4]. TS-1 contained 232 chemicals. Of the 232 chemicals, 131 showed ER binding activity and used as TS-3. The remaining 101 chemicals did not show ER binding activity and were defined as ER non-binders in this study. TS-1 was used to develop prediction models of ER binding activity [7,8,9]. TS-2 contained 1086 chemicals whose estrogenic activity data were curated from the literature. Of the 1086 chemicals, 350 were categorized as ER binders and 736 as ER non-binders [6]. The 350 chemicals and their logRBA values were used as TS-4.

### 2.3. Sunscreen Ingredients

The chemicals, as seen in Figure 1, are common ultraviolet (UV) filters used worldwide (1–24). They are considered to be “active” ingredients for they function as UV filters of either UVA and/or UVB rays and are thus “workhorse” ingredients in sun protection products. The remaining compounds were chosen as reference (25 and 27) or as examples of potential excipients found in sunscreen products and cosmetics (26, 28–38). The names shown for all these chemicals in Table 1 and Table 2 are both their United States adopted names and International Nomenclature of Cosmetic Ingredients (INCI) names.

### 2.4. Molecular Descriptors

Molecular descriptors are numerical descriptions of chemical structures and are used in quantitative structure-activity relationship (QSAR) models. The Mold2 descriptors calculation tool (http://www.fda.gov/ScienceResearch/BioinformaticsTools/Mold2/default.htm) [10] was used to generate the descriptors for the chemicals of the training sets (TS-1, TS-2, TS-3 and TS-4) as well as the sunscreen ingredients. Mold2 is a free software package that calculates molecular descriptors based on two-dimensional structures of chemicals. Mold2 is utmost fast in computation because it utilizes the extremely fast ring structure recognition algorithm [11] and adopted the efficient system of representation of chemical structures [12,13] that were initially developed in a structure elucidation expert system based on infrared [14] and nuclear magnetic resonance (NMR) spectra [15,16,17]. Mold2 molecular descriptors have been used in the development of various successful QSAR models [18,19,20,21,22]. For each chemical in the training data sets and the sunscreen ingredients, Mold2 calculated 777 molecular descriptors. The 777 molecular descriptors were then preprocessed by filtering out those with the same values for all the chemicals in a training data set. The descriptors, after filtering, were used to develop the DF classification and regression models.

### 2.5. Individual Models

Decision Forest (DF) is a machine learning algorithm that combines numerous decision tree models to make more accurate predictions [23,24,25]. DF can be used for development of both classification and regression models. Because combining multiple similar decision tree models most likely does not improve predictions, DF algorithm combines decision tree models that are well trained based on different chemical features. The diversity in chemistry of the decision tree models to be combined warrants that each of the combined models contributes to the final model in different but complementary ways. Our previous publications described the rationale and technical details of DF algorithm [23,24,25]. Briefly, DF algorithm consists of four steps: (1) construct a decision tree based on a pool of molecular descriptors; (2) exclude the descriptors used in the decision tree from the pool of molecular descriptors; (3) repeat steps (1) and (2) until improvement is seen in a sufficient number of decision trees that have been constructed; and (4) combine the results from all decision trees as the final predictions. 

In the development of DF classification models, each member decision tree model was assigned a probability between 0 and 1 to classify a chemical. The probability value was computed as division of number of ER binders in the terminal node by size of the terminal node. The probability values output from all the decision trees for a chemical were averaged to classify the chemical as ER binder, if the average probability >0.5, or ER non-binder if the average probability ≤0.5. To construct a DF regression model, a number of regression tree models were built. Each regression tree model output a regressed logRBA value for a chemical. The regressed logRBA values from all member regression trees were averaged as the final estimation of estrogenic activity for the chemicals. 

### 2.6. Prediction Performance

Performance of a DF classification model can be measured using different metrics. We used five parameters to measure performance of DF classification models: prediction accuracy, sensitivity, specificity, Matthews correlation coefficient (MCC) and balanced accuracy. These five performance parameters were calculated using Formulas (1) to (5):

(1) $$Accuracy=\frac{TP+TN}{TP+TN+FP+FN}$$

(2) $$Sensitivity=\frac{TP}{TP+FN}$$

(3) $$Specificity=\frac{TN}{TN+FP}$$

(4) $$MCC=\frac{TP\times TN-FP\times FN}{\sqrt{\left(TP+FP)(TP+FN)(TN+FP)(TN+FN\right)}}$$

(5) $$Balanced\;Accuracy=\frac{TP(TN+FP)+TN(TP+FN)}{2(TP+FN)(TN+FP)}$$

In Equations (1) to (5), true positive (*TP*) indicates number of ER binders that were classified as ER binders, true negative (*TN*) represents number of ER non-binders that were classified as ER non-binders, false negative (*FN*) means number of ER binders that were classified as ER non-binders, and false positive (*FP*) denotes number of ER non-binders that were classified as ER binders.

To measure performance of a DF regression model, the predicted values were compared with the actual values. The predictive ability of a DF regression model was quantified using the predictive squared correlation coefficient *Q^2^* calculated by Equation (6):

(6) $$Q^{2}=1-\frac{PRESS}{TSS}=1-\frac{\sum_{i=1}^{n}{\left(Y_{i}-P_{i}\right)}^{2}}{\sum_{i=1}^{n}{\left(Y_{i}-{\bar{Y}}_{i}\right)}^{2}}$$

In Equation (6), *n* is the total number of chemicals predicted using the model, the total sum of squares (*TSS*) is the sum of squared deviations from the mean of actual data, the sum of squares of the prediction errors (*PRESS*) is the sum of squared difference between predicted values and actual values. *P_i_* is the predicted value for chemical *i*, Y*_i_* is the actual value of chemical i, ${\bar{Y}}_{i}$ is the mean values of actual data of all chemicals predicted. The value of *Q^2^* is between −1 and 1. *Q^2^* = 0 indicates a model is not better than the prediction using the mean value of the actual data, i.e., the larger the *Q^2^*, the better the performance of the model.

### 2.7. Cross Validations

To evaluate the performance of DF classification and regression models, we conducted 5-fold cross validations on the four training data sets TS-1 and TS-2 as shown in Figure 2 and TS-3 and TS-4 as shown in Figure 3. More specifically, in one 5-fold cross validations run, a data set (TS-1 or TS-2 or TS-3 or TS-4) were randomly divided into five most equal portions. One portion was left and the other 4 portions were used as a training data set to construct a DF classification or regression model. The left portion of chemicals was then predicted using the constructed DF model. This process was iterated so that each of the 5 portions was left out once, and only once, to be predicted by the models trained using the remaining 4 portions. The results yielded from the 5 DF models were then combined for calculation of the above described performance parameter. The 5-fold cross validation process was repeated 1000 times using different random divisions of the whole data set into five most equal portions so that the performance estimation is a statistically robust estimation. 

### 2.8. External Validations

Result from a 5-fold cross validation consists of predictions for all chemicals in a data set and usually is better than the result of testing on a new data set. External validation is more reliable and necessary performance evaluation of a QSAR model. In this study, most chemicals in TS-2 were not included in TS-1 and were used to estimate performance of the DF classification model M-1 that was trained using TS-1 as the external validation as shown in Figure 2. Similarly, most chemicals in TS-4 were not included in TS-3 and were used to estimate performance of the DF regression model M-3 that was trained using TS-3 as the external validation as shown in Figure 3.

### 2.9. Consensus Modeling

Multiple factors affect performance of QSAR models. For example, limited chemical structural space covered by the training chemicals makes the model performing worse on chemicals out of the chemical space. To improve model performance, a consensus strategy was adopted for the prediction of ingredient estrogenic activity. 

In the consensus classification modeling, each ingredient was classified into ER binder or non-binder using the two DF classification models (M-1 and M-2) that were constructed using TS-1 and TS-2. The probabilities output from M-1 and M-2 indicate how likely the ingredient can be classified as ER Binder. The two probabilities of the same ingredient were averaged as the consensus classification (ER binder or non-binder) for the ingredient.

In the estimation of ER binding affinity by the consensus regression modeling for the ingredients that were classified as ER binders, the two DF regression models (M-3 and M-4) that were constructed using TS-3 and TS-4 were used to predict logRBA values for the ingredients. The predicted logRBA values from M-3 and M-4 for the same ingredient were averaged as the consensus prediction for the ingredient.

### 2.10. Prediction Confidence

The classification (binder or non-binder) output from the consensus classification modeling for a chemical is a probability *p* that is a continuous value and was used as the likelihood to classify the chemical as an ER binder (*p* > 0.5) or non-binder (*p* ≤ 0.5). This *p* value indicates the confidence for the classification. A good classification model is expected to have more chemicals classified at high confidence level. The classification confidence was calculated for each of the ingredients from the consensus DF classification modeling using Equation (7):

(7) $$Confidence=\frac{\left|p-0.5\right|}{0.5}$$

The calculated classification confidence is a value between 0 and 1. The larger the confidence value is, the more reliable is the classification. 

## 3. Results 

### 3.1. Database Search

We first searched EDKB for estrogenic activity data of 27 ingredients of interest, including 24 UV filters, 2 reference compounds and 1 sunscreen product excipient. Experimental estrogenic data were found for 5 UV filters and the search results are listed in Table 1. The experimental ER binding affinities in Table 1 were given as logarithmic relative binding affinity (logRBA) values to the nature hormone estradiol whose logRBA was set to 2. Aminobenzoic acid (**1**) had weak estrogenic activity in yeast two-hybrid assay with a logarithmic 10% relative activity (logRA10) of −3.523 that was calculated by the concentration of a chemical showing 10% of the agonist activity of 10^−7^ M of the natural hormone E2, which is the optimum effective concentration for E2 [26]. Compounds **3** (oxybenzone) and **4** (dioxybenzone) were determined as ER non-binders using rat uterine cytosolic ER competitive binding assay [5]. Compounds **2** (octisale), **4** (dioxybenzone), and **5** (padimate) did not show activity in ER in vitro binding assay with RI-labeled estradiol as reference ligand [27]. We were not able to find experimental data for the rest of the 19 UV filters in EADB. The reference compound triclosan (**25**) showed weak ER binding activity in rat uterine cytosolic ER competitive binding assay with a logRBA value of −3.280 [28].

### 3.2. Cross Validations

**Figure 4:**
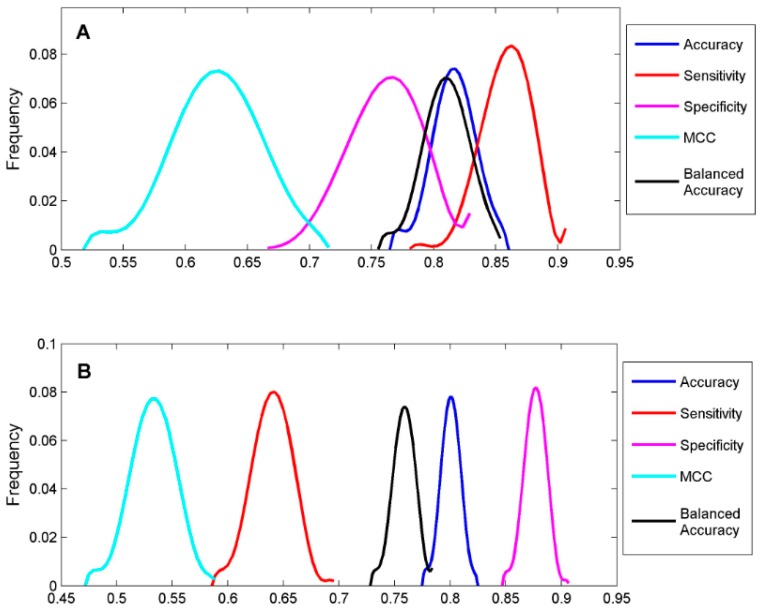
Classification performance of the 5-fold cross validations. Classification accuracy (blue), sensitivity (red), specificity (magenta), MCC (cyan) and balanced accuracy (black) of the 1000 iterations of 5-fold cross validations were plotted for TS-1 (**A**) and TS-2 (**B**). Parameter values were indicated at the *x*-axis and the *y*-axis represents the frequency of cross validations.

To classify the 19 sunscreen ingredients into ER binders and non-binders, we built two DF classification models using two TS-1 and TS-2. To assess if reliable classification models can be constructed, 1000 iterations of 5-fold cross-validations were conducted on TS-1 and TS-2 for estimation performances of the DF classification models. The five performance parameters that were calculated using formulas (1) to (5) for the 1000 iterations of 5-fold cross validations were plotted for TS-1 in Figure 4A and TS-2 in Figure 4B. The mean values and standard deviations of the five performance parameters for TS-1 and TS-2 are listed in Table 3. The DF classification models had overall classification accuracy of >80%, indicating good classification models could be generated using TS-1 and TS-2. The small standard deviations for the performance parameter values demonstrated that the DF classification models performed consistently. Therefore, the classification models (M-1 and M-2) built from TS-1 and TS-2 should be statistically reliable.

**Figure 5:**
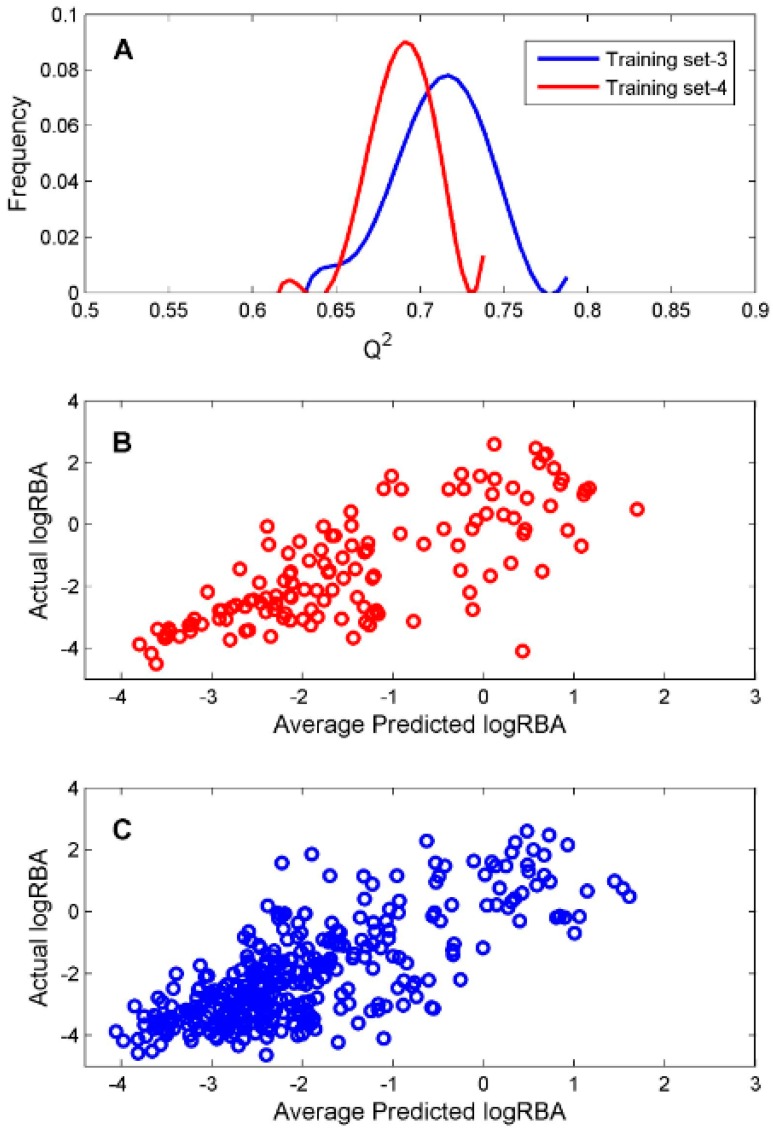
Regression performance of the 5-fold cross validations. The distributions of *Q^2^* values were plotted as blue and red lines for TS-3 and TS-4 (**A**). The average predicted logRBA of the 1000 iterations of 5-fold cross validations were plotted against the actual logRBA for TS-3 (**B**) and TS-4 (**C**).

For the ingredients that were classified as ER binders by the consensus DF classification model, we constructed two DF regression models using two TS-3 and TS-4 for estimation of their binding affinity. To evaluate reliability of the constructed regression models, 1000 iterations of 5-fold cross-validations were conducted on TS-3 and TS-4 for estimation performances of the DF regression models. The predictive squared correlation coefficient *Q^2^* values of the 1000 iterations of 5-fold cross validations were plotted as blue and red lines in Figure 5A for TS-3 and TS-4, respectively. 

The mean and standard deviation of the *Q^2^* values were 0.712 and 0.027 for TS-3 and 0.690 and 0.018 for TS-4, indicating accurate and robust regression models could be generated based on TS-3 and TS-4. The predicted logRBA values of the 131 chemicals in TS-3 were plotted against their actual experimental logRBA values in Figure 5B. The predicted logRBA values of the 350 chemicals in TS-4 were plotted against their actual experimental logRBA values in Figure 5C. Overall, the predicted ER binding affinity values were close to the actual binding affinity values for the 5-fold cross validations on TS-3 and TS-4. Therefore, the regression models (M-3 and M-4) trained on TS-3 and TS-4 should be statistically reliable.

### 3.3. External Validations

Most of the chemicals in TS-2 were not included in TS-1. Those chemicals were used as an external data set to validate the DF classification model that was built from TS-1. The DF classification model M-1 was first trained using TS-1. After filtering, 81 Mold2 molecular descriptors were used to develop M-1. M-1 was then used to classify the chemicals from TS-2. The classification model M-1 yielded accuracy 0.770, sensitivity 0.803, specificity 0.754, MCC 0.527, and balanced accuracy 0.779. Compared with the performance of 5-fold cross validations shown in Table 3, the external validations had similar performance to the cross validations, further indicating that reliable classifications could be achieved with the DF classification models trained on TS-1 and TS-2.

**Figure 6:**
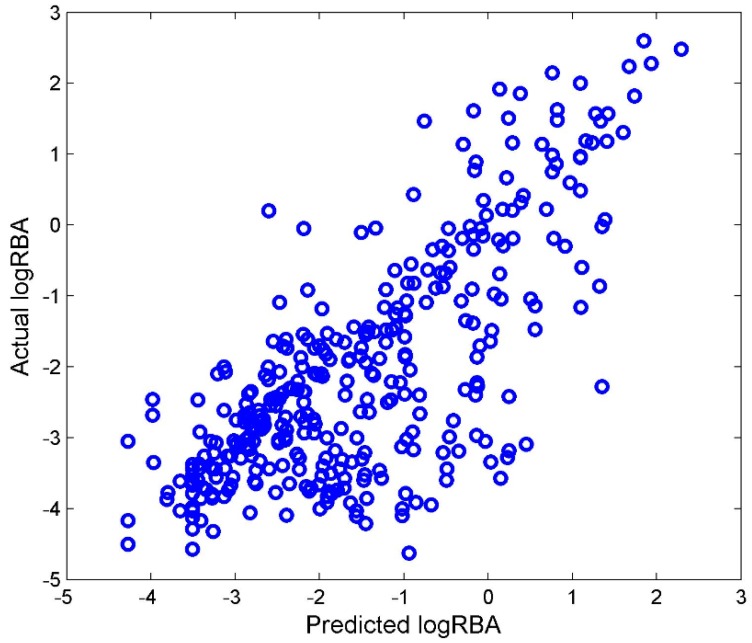
External validation results. The *x*-axis indicates actual logRBA values and the *y*-axis gives the estimated logRBA values from model M-3 that was developed using TS-3.

Most of the chemicals in TS-4 were not included in TS-3. Those chemicals were used as an external data set to validate the DF regression model that was built from TS-3. The DF regression model M-3 was first constructed using TS-3. After filtering, 105 Mold2 molecular descriptors were used to develop M-3. M-3 was then used to quantitatively estimate ER binding affinity for the chemicals from TS-4. The predicted logRBA values from regression model M-3 were plotted against with the actual logRBA values for the external testing chemicals in Figure 6. 

Compared with the performance of 5-fold cross validations shown in Figure 5A, the external validations had similar performance to the cross validations, *Q^2^* = 0.744. Therefore, TS-3 and TS-4 could be used to develop reliable regression models. 

### 3.4. Consensus Modeling

We used a consensus modeling strategy to qualitatively and quantitatively predict the estrogenic activity (classify sunscreen ingredients into ER binder or non-binder and estimate ER binding affinity of predicted ER binders) for the 19 sunscreen ingredients for which experimental estrogenic activity data were not found in EADB. As shown in Figure 2, DF classification models, M-1 and M-2, were first generated using TS-1 and TS-2, respectively. The classification models were then used to calculate the probabilities of the 19 sunscreen ingredients to classify them as ER binders or non-binders. The two probabilities for a sunscreen ingredient were averaged to make a consensus classification of the compound as an ER binder or non-binder. The results of the consensus classification modeling of the 19 sunscreen ingredients are shown in Table 1. Of the 19 sunscreen ingredients, only two were classified as ER binders, while the remaining 17 were classified as ER non-binders by the consensus classification model.

The probability for a sunscreen ingredient to be classified as ER binder from the consensus classification modeling was converted to provide confidence for classification of the compound using Formula (7). The confidence values of the 19 sunscreen ingredients are listed in Table 1. 

For the two sunscreen ingredients that were classified as ER binders, their ER binding affinity values were further estimated using the DF regression models M-3 and M-4 that were developed based on TS-3 and TS-4, respectively. The regression models were then used to estimate ER binding affinity (logRBA) values for the 2 sunscreen ingredients. The two estimated logRBA values for a sunscreen ingredient were averaged to make a consensus estimation of logRBA for the compound. The logRBA values of the consensus classification modeling for the two sunscreen ingredients are listed in Table 1. Both sunscreen ingredients had low logRBA values.

Ketoconazole (**24**), another reference compound known to lack estrogen activity and butyloctyl salicylate (**26**), a sunscreen product excipient with unknown estrogen activity were also included in the analysis. The latter was predicted to be an ER binder and both are listed in Table 1.

Interestingly, the prediction for sulisobenzone (**6**) was not consistent with that of other benzophenone derivatives, i.e., oxybenzone (**3**) and dioxybenzone (**4**). This led to the predictions being extended to include other benzophenone derivatives and in turn, to look for a potential explanation based on chemical structure. In addition to their use as UV filters in sunscreen products, benzophenones are typically used as light stabilizers (or photostabilizers) in personal care products and fragrances. Light stabilizers are added to protect these products from chemical or physical deterioration induced by light. This function has also made benzophenones suitable for other products, such as plastic surface coatings for food packaging.

Table 2 shows the predictions by the consensus DF classification model of ER binder activity for benzophenone derivatives known to be used in consumer products. In addition to compound **6**, compounds **28**, **29**, **30** and **31** were predicted to be ER binders. 

From a structural point of view, the results obtained can be explained by the presence of a 4-hydroxyl and phenolic groups (i.e., compounds **28**, **29**) [7]. Chemicals with suitable molecular weight are expected to fit the binding pocket of ER and an electronegative atom or group increases binding interaction with ER (i.e., compounds **6**, **30** and **31**) [29]. 

## 4. Discussion

Timely go/no-go decisions on ingredients to add to a formulation is key for consumer products such as sunscreens that generally contain multiple UV filters and excipient ingredients, both influenced by a fast pace of innovations. Computational tools using structure-activity relationships can be used by stakeholders to screen out and abandon prospective ingredients after they are predicted to possess an undesirable activity, such as estrogen receptor binding. This will help direct resources for more definitive but expensive data-driven testing only to those ingredients not predicted to have the undesirable activity, in addition to help reduce unnecessary animal testing. 

In our previous study, we developed a tree-based model using the same training data set (TS-1) for predicting ER binding activity of more than 50,000 environmental chemicals [7]. Compared to our previous model that had 87.9% accuracy in the training and 0.526 of MCC value from validation on the 463 chemicals experimentally tested by Nishihara et al. [26], the DF model trained in this study had an improved training accuracy of 97%. The model was validated using a larger data set with 1086 chemicals and yielded a slightly better MCC value of 0.527.

Of the 32 chemicals with unknown ER binding activity in EDKB that were evaluated in this work, seven were predicted to be active estrogenic compounds and the remaining 25 were predicted to be inactive ones. It should be noted that the predictions made are devoid of consideration of possible metabolic activation/deactivation of the compounds but, on the positive side, there are no (trace) impurities/contaminants that may influence the results. Recognizing its advantages and limitations, the following seven potentially active estrogenic compounds were identified: benzophenone-4 (**6**) benzophenone-5 (**30**), 4-methylbenzylidene camphor (**7**), benzophenone-1 (**28**), benzophenone-2 (**29**) and benzophenone-7 (**31**). Post-prediction literature surveying estrogenic experiment data of the compounds supported our model predictions. The estrogenic activity data have been reported for benzophenone-4 (**6**) [30,31,32], 4-methylbenzylidene camphor (**7**) [33], benzophenone-1 (**28**) [30,31] and benzophenone-2 (**29**) [30,31,34,35,36]. As a result, all of these ingredients pose a concern that they may be potential endocrine-mediated health hazards. If stakeholders find these ingredients to be essential for the product formulations, priority should be given to the gathering of scientific evidence in support of the safe use of these ingredients at levels experienced by human populations and that is consistent with cumulative exposures to products containing any of these compounds.

The training data sets used in this study contain the data obtained from an assay that used mixture of two subtypes of ER (ERα and ERβ). The ER used in the assay was extracted from uterine cytosol of non-pregnant Sprague-Dawley rats. Briefly, after rats were sacrificed by CO_2_ asphyxiation, uteri were excised, trimmed of excess fat and mesentery. Uterine tissue was homogenized and transferred to pre-cooled ultracentrifuge tubes and centrifuged. After centrifugation, the ER-rich supernatant was used in the competitive assay. This assay was used to measure binding activity to ER, but not specific to ERα or ERβ or both. Therefore, the model developed in this study is unable to predict binding activity to specific subtypes of ER. 

Plans for future work include widening the reported approach to a much larger number of sunscreen product excipients and extending the model to also make predictions on the androgen receptor activity of all these ingredients. The value of these predictions may be enhanced as basic research continues to elaborate on the understanding of hormonal signaling pathways and related disorders.

## 5. Conclusions

To conclude, in the absence of relevant scientific data, the application of predictive computational models as reported here represents a step forward in characterizing the level of concern with estrogen receptor activity among ingredients in widely used consumer products such as sunscreens. The model presented is essentially a risk assessment tool and its predictions do not pre-empt the regulatory conclusions that may eventually be made on the basis of experimental data as it becomes available. Taken together, the intent is for this model to lead to safer drugs in order to protect and promote public health.

## Figures and Tables

**Table 1:** Estrogenic activity of sunscreen ingredients.

Compounds, UV Filters			Qualitative Prediction *		Quantitative Prediction **
ID	CAS	OTC Drug Name/*INCI Name*	+/−	Conf	
1	150-13-0	Aminobenzoic acid/*PABA*	+	(26)	logRA10 = −3.523
2	118-60-5	Octisale/*Ethylhexyl Salicylate*	−	(30)	
3	131-57-7	Oxybenzone/*Benzophenone-3*	−	(5)	
4	131-53-3	Dioxybenzone/*Benzophenone-8*	−	(5,27)	
5	21245-02-3	Padimate/*Penthyl Dimethyl PABA*	−	(27)	
6	4065-45-6	Sulisobenzone/*Benzophenone-4*	+	0.310	−2.915
7	38102-62-4	*4-Methylbenzylidene camphor*	+	0.102	−1.431
8	2174-16-5	Trolamine salicylate/*TEA salicylate*	−	0.794	
9	27503-81-7	Ensulizole/*Phenylbenzimidazole Sulfonic Acid*	−	0.034	
10	70356-09-1	Avobenzone/*Butyl Methoxydibenzoylmethane*	−	0.601	
11	104-28-9	Cinoxate/*Cinoxate*	−	0.654	
12	134-09-8	Meradimate/*Menthyl Anthranilate*	−	0.581	
13	5466-77-3	Octinoxate/*Octyl methoxycinnamate*	−	0.597	
14	6197-30-4	Octocrylene/*Octocrylene*	−	0.157	
15	71617-10-2	*Isoamyl p-Methoxycinnamate*	−	0.654	
16	155633-54-8	*Drometrizole trisiloxane*	−	0.077	
17	103597-45-1	*Methylene bis-Benzotriazolyl Tetramethylbutylphenol*	−	0.191	
18	118-56-9	Homosalate/*Homosalate*	−	0.206	
19	13463-67-7	Titanium dioxide/*Titanium dioxide*	−	0.694	
20	1314-13-2	Zinc oxide/*Zinc oxide*	−	0.795	
21	154702-15-5	*Diethylhexyl butamidotriazone*	−	0.756	
22	88122-99-0	*Ethylhexyl triazone*	−	0.756	
23	187393-00-6	*bis-Ethylhexyloxyphenol methoxyphenyl triazine*	−	0.012	
24	92761-26-7	Ecamsule/*terephthalylidene dicamphor sulfonic acid*	−	0.072	
	**Reference Compounds, non UV filters**				
25	3380-34-5	Triclosan/*Triclosan*	+	(28)	logRBA = −3.280
26	190085-41-7	*Butyloctyl Salicylate*	+	0.827	−0.853
27	65277-42-1	Ketoconazole	−	0.046	

* Prediction confidence is a number between 0 and 1 for indication of confidence for a prediction: the smaller the number, the less confident the prediction. ** Prediction is in log10(RBA). RBA is the relative binding affinity to the natural estrogen, estradiol. RBA of estradiol is set to 100 and, thus, its log10(RBA) = 2. INCI = International Nomenclature of Cosmetic Ingredients. INCI names are used in the United States, the European Union, Japan and many other countries for listing ingredients on sunscreen product labels.

**Table 2:** Predicted Estrogenic activity of benzophenone derivatives.

Compounds, Benzophenone Derivative			Qualitative Prediction *		Quantitative Prediction **
ID	CAS	Name/*INCI Name*	+/−	Conf		
3	131-57-7	Oxybenzone/*Benzophenone-3*	−	(5)	
4	131-53-3	Dioxybenzone/*Benzophenone-8*	−	(5,27)	
6	4065-45-6	Sulisobenzone/*Benzophenone-4*	+	0.310	−2.915
28	131-56-6	2,4-dihydroxybenzophenone/*Benzophenone-1*	+	0.875	−2.710
29	131-55-5	2,2′,4,4′-Tetrahydroxybenzophenone/*Benzophenone-2*	+	0.900	−1.609
30	6628-37-1	Sulisobenzone sodium/*Benzophenone-5*	+	0.600	−2.614
31	85-19-8	5-Chloro-2-hydroxybenzophenone/*Benzophenone-7*	+	0.777	−2.778
32	131-54-4	2,2′-Dihydroxy-4,4′-dimethoxybenzophenone/*Benzophenone-6*	−	0.100	
33	76656-36-5	Sodium 2,2′-dihydroxy-4,4′-dimethoxybenzophenone-5,5′-disulfonate/*Benzophenone-9*	−	0.300	
34	1641-17-4	Mexenone, 2-hydroxy-4-methoxy-4′-methylbenzophenone/*Benzophenone-10*	−	0.100	
35	1341-54-4	*Benzophenone-11*	−	0.100	
36	1843-05-6	Octabenzone/*Benzophenone-12*	−	0.140	
37	954-16-5	Trimethylbenzophenone	−	0.997	
38	119-61-9	Benzophenone	−	0.996	

* Prediction confidence is a number between 0 and 1 for indication of confidence for a prediction: the smaller the number, the less confident the prediction. ** Prediction is in log10(RBA). RBA is the relative binding affinity to the natural estrogen, estradiol. RBA of estradiol is set to 100 and, thus, its log10(RBA) = 2. INCI = International Nomenclature of Cosmetic Ingredients. INCI names are used in the United States, the European Union, Japan and many other countries for listing ingredients on sunscreen product labels.

**Table 3:** Cross validation results.

Parameter	Result (Mean ± Std)	
	TS-1	TS-2
Accuracy	0.816 (±0.018)	0.801 (±0.009)
Sensitivity	0.859 (±0.020)	0.640 (±0.018)
Specificity	0.761 (±0.031)	0.877 (±0.010)
MCC	0.625 (±0.037)	0.533 (±0.021)
Balanced Accuracy	0.810 (±0.019)	0.758 (±0.010)

Std: standard deviation; MCC: Mathews correlation coefficient.
